# RPE With ROCK-Mediated Epithelial-Mesenchymal Transition as a Key Contributor of Subretinal Fibrosis in AMD

**DOI:** 10.1167/iovs.67.5.33

**Published:** 2026-05-14

**Authors:** Iori Wada, Keijiro Ishikawa, Muneo Yamaguchi, Shotaro Shimokawa, Ji Rui, Kenichiro Mori, Yoshihiro Kaizu, Mitsuru Arima, Shoji Notomi, Yusuke Murakami, Shigenori Inagaki, Yuta Kohro, Makoto Tsuda, Kensuke Shibata, Ryo Terao, Takeshi Terabayashi, Toshimasa Ishizaki, Shigeo Yoshida, Tatsuro Ishibashi, Susumu Ishida, Rajendra S Apte, Koh-Hei Sonoda, Shintaro Nakao

**Affiliations:** 1 Department of Ophthalmology, Graduate School of Medical Sciences, Kyushu University, Fukuoka, Japan; 2 Graduate School of Medical Sciences, Kyushu University, Fukuoka, Japan; 3 Department of Life Innovation, Faculty of Pharmaceutical Sciences, Kyushu University, Fukuoka, Japan; 4 Department of Microbiology and Immunology, Graduate School of Medicine, Yamaguchi University, Yamaguchi, Japan; 5 John F. Hardesty, MD Department of Ophthalmology & Visual Sciences, Washington University School of Medicine, St. Louis, Missouri, United States; 6 Department of Pharmacology, Oita University, Faculty of Medicine, Yufu, Japan; 7 Department of Ophthalmology, Kurume University School of Medicine, Kurume, Japan; 8 Laboratory of Ocular Cell Biology and Visual Science, Department of Ophthalmology, Faculty of Medicine and Graduate School of Medicine, Hokkaido University, Sapporo, Japan; 9 Department of Developmental Biology, Washington University School of Medicine, St. Louis, Missouri, United States; 10 Department of Medicine, Washington University School of Medicine, St. Louis, Missouri, United States; 11 Department of Ophthalmology, Juntendo University Graduate School of Medicine, Tokyo, Japan; 12 Juntendo He Advanced Ophthalmology Technology Laboratory, Tokyo, Japan

**Keywords:** VEGF, Rho-kinase, MET, retinal pigment epithelium, EndMT

## Abstract

**Purpose:**

To investigate the cellular origin and molecular mechanisms underlying subretinal fibrosis (SRF) in neovascular AMD (nAMD), with a focus on the role of RPE and Rho-associated coiled-coil-containing protein kinase (ROCK)–mediated epithelial-mesenchymal transition (EMT).

**Methods:**

Fate-mapping with lineage tracing, laser capture microdissection, microarray and RT-PCR analyses, immunohistochemistry, and functional assays with ROCK inhibitors (Ripasudil, Belumosudil) were used to assess EMT/endothelial-to-mesenchymal transition contributions to SRF and ROCK pathway involvement.

**Results:**

Fate mapping identified RPE and endothelial cells as sources of myofibroblasts. EMT-related gene upregulation and ROCK1/2 expression were observed in SRF lesions. RPE-specific ROCK2 knockout significantly reduced fibrosis and EMT markers. Ripasudil suppressed SRF development and reversed EMT both in vivo and in vitro. ROCK expression was confirmed in human choroidal neovascularization membranes.

**Conclusions:**

RPE-derived myofibroblasts via ROCK2-mediated EMT play a dominant role in SRF formation. ROCK inhibitors, particularly Ripasudil, may serve as effective therapeutic agents against fibrosis in nAMD.

Neovascular AMD (nAMD) is a progressive, degenerative chronic inflammatory disease in elderly populations by the development of macular neovascularization (MNV) and is the leading cause of vision loss in developed countries.^1^^,^^2^ Although clinically applied anti- VEGF therapy is widely used as the first-line treatment for MNV and the associated exudative disorders with nAMD,^3^^–^^5^ long-term clinical studies revealed that even in patients with improved visual acuity during the first two years of anti-VEGF therapy, visual gains were not maintained after five years.^6^^–^^8^ A cohort study has identified several risk factors for vision loss, including subretinal fibrosis (SRF), macular geographic atrophy, macular scarring, MNV in the macula, and subretinal hyperreflective material.^9^ Moreover, examination of data from a neovascular age-related macular degeneration registry showed that, even after 10 years of anti-VEGF treatment, approximately 40% of eyes still exhibited SRF. Notably, the incidence of SRF was also found to increase with longer treatment duration.^10^ Although a number of studies have identified the molecular regulators of MNV,^11^^–^^16^ how fibrosis develops in the sub-retinal space remains a significant knowledge gap, and the lack of treatments for SRF represent a significant therapeutic challenge.

Fibrosis is regulated by controlling myofibroblast differentiation, mobilization, proliferation and activation in chronic diseases similar to nAMD, such as pulmonary fibrosis and kidney disease.^17^ Myofibroblasts also produce extracellular matrix (ECM), such as type 1 collagen, which is involved in forming fibrosis.^17^ Identifying the origin of myofibroblasts is essential in the treatment of fibrosis. Various reports investigate the origin of myofibroblasts in organs where fibrosis can develop, such as kidney, lung, and liver.^18^^–^^21^ In ocular diseases, a previous immunohistochemical study with human tissue showed that SRF in AMD contains RPE, vascular endothelium, inflammatory cells, myofibroblasts, and ECM.^22^ Although RPE, choroidal pericytes, glial cells, fibroblasts, and macrophages are suggested as the potential sources of myofibroblasts in SRF,^23^^,^^24^ the origin has not been identified.

Epithelial-mesenchymal transition (EMT) and endothelial-to-mesenchymal transition (EndMT) are also essential for the differentiation into myofibroblasts in the development of fibrosis.^25^ EMT is involved in embryonic development and wound healing, whereas its overactivation in chronic diseases such as AMD promotes pathological tissue fibrosis.^17^^,^^26^ The RPE detachment and dissociation process during MNV formation is necessary to initiate the EMT program.^27^ RPE cells undergo EMT, leading to the loss of their cell adhesions and apical-basal polarity.^28^ Similarly, disruption of vascular endothelial cell-cell contact is required to initiate the EndMT program in models of organ fibrosis.^29^ Additionally, numerous extracellular ligands participate in triggering and advancing EMT and EndMT programs. Serving as a key regulator in the control of both EMT and EndMT, the TGF-β ligand is considered a pivotal controller of these processes.^30^^,^^31^

In this study, we focused on EMT and EndMT to directly investigate the origin of myofibroblasts involved in SRF development in AMD. By combining microarray analysis with laser microdissection, we identified SRF-specific mechanisms and demonstrated that Rho-associated coiled-coil-containing protein kinase (ROCK) may serve as a novel therapeutic target for SRF in nAMD.

## Methods

### Experimental Design

#### Animals

We generated *BEST1* and *VE-cadherin*-specific *ROCK1*- and *ROCK2*-cKO mice, *ROCK1^flox/flox^* mice or/and *ROCK2^flox/flox^* mice were crossed with *BEST1-Cre* and *VE-cadherin-Cre* mice, respectively. The resulting offspring, *BEST1-Cre; ROCK1^flox/flox^ROCK2^flox/flox^* mice, *VE-cadherin-Cre; ROCK1^flox/flox^ROCK2^flox/flo^*, and their littermates were also used for experiments.

#### Laser-Induced Choroidal Neovascularization Model

Male mice, aged between seven to nine weeks old, were used in all studies to eliminate sex-related bias in the size of laser-induced CNV lesions. Male C57BL/6J mice that do not carry the rd8 and Pde6brd1 mutations were purchased from the Jackson Laboratory (The Jackson Laboratory, Bar Harbor, ME, USA). Choroidal neovascularization (CNV) mouse models were generated as described previously.^60^^,^^61^ To evaluate the effect of ROCK inhibition and anti-VEGF therapy on SRF formation, intravitreal injections were administered beginning on day 14 after laser induction, a time point chosen to minimize the influence of CNV-related EndMT, because CNV regression has been reported to occur around this stage. Each mouse eye received a 1-µL intravitreal injection using a 33-gauge Hamilton syringe. Treatment groups included balanced salt solution (BSS; control); Fasudil (30 µM; a ROCK inhibitor; Wako Pure Chemical Industries, Osaka, Japan); Ripasudil (3 or 30 µM; Kowa Company, Ltd., Nagoya, Japan); and Ripasudil (30 µM) combined with Aflibercept (Eylea; Regeneron, Tarrytown, NY, USA; 40 mg/mL formulation). Injections were performed on days 14, 17, 20, 23, and 26 post-laser.

#### Immunofluorescence Staining of Mouse Ocular Tissues

Mouse eyes were enucleated and fixed with 4% paraformaldehyde for 30 minutes at 4°C. For whole-mount preparation, the retinas were microsurgically exposed by removing other portions of the eye. Tissues were placed in methanol for 20 minutes and washed with Tween PBS once for 15 minutes. Tissues were incubated overnight at 4°C with anti-mouse pan-CK (sc-8018; Santa Cruz, Dallas, TX, USA; 1:200), anti-mouse CD31 mAB (550274; BD Pharmingen, San Diego, CA, USA; 1:100), anti-rabbit type1 collagen (35429; Rockland, Rockland, NY, USA; 1:100), and anti-goat αSMA mAb (ab21027; Abcam, Boston, MA, USA; 1:200) diluted in PBS containing 10% goat serum and 1% Triton X-100. Tissues were washed four times for 15 minutes each in Tween PBS followed by incubation with secondary fluorescent antibodies (Alexa Fluor; Thermo Fisher Scientific, Waltham, MA, USA; 1:200) overnight at 4°C. The flat mounts were prepared on glass slides using mounting medium (TA-030-FM Mountant Permafluor; Lab Vision Corporation, Fremont, CA, USA). The flat mounts were examined by fluorescence microscopy (Leica TCS SP2 laser scanning confocal microscope; Leica Microsystems GmbH, Wetzlar, Germany). Frozen sections of harvested choroid embedded in optimal cutting temperature medium and 2% sucralose in PBS (2:1) were immunostained using antibodies to pan-CK (sc-8018; Santa Cruz; 1:200), CD31 (550274; BD Pharmingen; 1:100), αSMA (ab21027; Abcam; 1:200), type1 collagen (35429; Rockland; 1:100), ZO-1 (61–7300; Thermo Fisher Scientific; 1:200), and secondary fluorescent antibodies (Alexa Fluor, 1:200). Pictures were taken using fluorescence microscopy (Leica TCS SP2 laser scanning confocal microscope; Leica Microsystems GmbH). Five random fields of view were photographed to quantify immunostained or fluorescent images, and the number of immunolabeled cells was counted per × 40 field of view.

#### Quantification of BEST1⁺/αSMA⁺ and VE-Cadherin⁺/αSMA⁺ Double-Positive Areas in Subretinal Fibrosis

Subretinal fibrosis sections obtained from BEST1-Cre-tdTomato and VE-cadherin-Cre-tdTomato mice were subjected to immunofluorescence staining for αSMA. Images were captured using a fluorescence microscope under identical acquisition settings. Quantitative analysis was performed using ImageJ (NIH, Bethesda, MD, USA). The merged areas of BEST1⁺/αSMA⁺ and VE-cadherin⁺/αSMA⁺ signals were identified by thresholding each fluorescence channel and calculating the overlapping regions. The double-positive area was defined as the merged area of lineage-traced cells (tdTomato) and αSMA signals and was expressed as a percentage of the total image area.

#### Laser Capture Microdissection

Slides were thawed, and tissues were fixed with acetone for three minutes and washed with diethyl pyrocarbonate water for 60 seconds. Laser capture microdissection (Carl Zeiss Meditec, Jena, Germany) was applied. In brief, a microbeam was used to cut tissues, and a laser catapult was applied to collect the tissues into collecting tubes. RNA was extracted with the Qiagen RNeasy Micro kit (74004; Qiagen, Venlo, Netherlands), and the quality and quantity were assessed with Bioanalyzer RNA pico chips. RNA amplification and cDNA synthesis were performed with Ovation PicoSL WTA System V2 (NUG-3302-12; NuGen Technologies Inc., Chicago, IL, USA) according to the manufacturer's instructions, and the Qiagen MinElute Reaction Cleanup Kit was applied for cDNA purification (28204; Qiagen).

#### Gene Expression Analysis

According to the manufacturer's instructions, the total RNA was isolated from cells using RNeasy Mini Kit (Qiagen). RNA samples were quantified by an ND-1000 spectrophotometer (NanoDrop Technologies, Wilmington, DE, USA), and the quality was confirmed with a 2200 TapeStation (Agilent Technologies, Santa Clara, CA, USA). The cRNA was amplified, labeled with total RNA using GeneChip WT Pico Kit (Thermo Fisher Scientific), and hybridized to Thermo Fisher Scientific Clariom D Assay mouse according to the manufacturer's instructions. An Affymetrix scanner (Affymetrix, Santa Clara, CA, USA) scanned all hybridized microarrays. Relative hybridization intensities and background hybridization values were calculated using the Affymetrix Expression Console (Affymetrix). The raw signal intensities of all samples were normalized with the SST-RMA algorithm (gene level) using Affymetrix Expression Console 1.4.1 software. To identify up- or down-regulated genes, we calculated Z-scores as previously reported^61^ and ratios (non-log scaled fold-change) from the normalized signal intensities of each probe for comparison between control and experiment samples. Then we established criteria for regulated genes: (up-regulated genes) Z-score ≥ 2.0 and ratio ≥ 1.5-fold, (down-regulated genes) Z-score ≤ −2.0 and ratio ≤ 0.66.

#### Real-Time Quantitative PCR

All mice were anesthetized by intraperitoneal injection of 15 mg/kg ketamine and 7 mg/kg xylazine. Total RNA of retinal tissues or cultured cells was extracted using a NucleoSpin RNA Kit (Macherey-Nagel, Duren, Germany). Cultured cells were incubated with Ripasudil or Belumosudil (HY-15307, MedChemExpress, Monmouth Junction, NJ, USA) at different concentrations (0.3, 3, 30 µM) in their respective complete media containing 10 ng/mL TGF-β2. Total RNA was reverse transcribed using a First Strand cDNA Synthesis Kit for RT-PCR (Roche, Basel, Switzerland). The real-time quantitative PCR (qPCR) was performed with SYBR Premix Ex Taq (Takara Bio Inc, Shiga, Japan) using a LightCycler 96 (Roche).

#### CNV and Subretinal Fibrosis Volume Measurement

After laser photocoagulation, the CNV and subretinal fibrosis volumes were measured in choroidal whole mounts on days 14, 21, and 28. The CNV is reduced by day 14 after laser photocoagulation referenced in the previous report.^36^ Mouse whole eyes were fixed in 4% paraformaldehyde at 4° for 30 minutes. And then eyecups were made and fixed with methanol (on ice for 20 minutes). After fixation, CD31, type1 collagen, and αSMA antibody were added to the mouse eyecup and incubated at 4° overnight to detect CNV, subretinal fibrosis, and EMT-associated cells. Alexa Fluor was the secondary antibody against CD31, type1 collagen, and αSMA. Samples were coverslipped and examined by using the confocal microscope. Fluorescence volume measurements were recorded by generating image stacks of optical slices within lesions.

#### Histological Analysis

The eyes were excised to measure the fibrosis area in laser-induced CNV model. Each slice was then fixed in 10% buffered formalin for four hours, embedded in paraffin, and cut into 30-µm-thick sections with a microtome. These sections were stained with Masson-Trichrome (Muto Pure Chemicals Co., Tokyo, Japan) and Sirius red (Sigma-Aldrich, St. Louis, MO, USA).

#### Time-Lapse Imaging System

Time-lapse images were captured using an inverted fluorescence microscope (Keyence BZ-X700; Keyence, Osaka, Japan). Time-lapse observation was started 24 hours after seeding the cells to allow hRPE to adhere to the bottom of the dish. HRPE in the 24-well dish was cultured in 500 µL of Dulbecco's modified Eagle medium supplemented with 10 ng/ml TGF-β2 and treated with Ripasudil (30 µM) 24 hours after the TGF-β2 stimulation. Time-lapse images were obtained every 30 minutes for two days. The most focused images were selected using the analysis application BZ-II Analyzer (Keyence), and time-lapse continuous images were acquired.

#### Proliferation Assay

Subconfluent hRPE were trypsinized and were seeded at 37°C in coated 96-well plates at an initial density of 3 × 10^3^ cells per well in a 100 µL complete medium. The following day, cells were incubated with Ripasudil at different concentrations (0.3, 3, 30 µM) in their respective complete media. In a serum-free medium, hRPE was stimulated with 10 ng/mL TGF-β2 (rhVEGF165; R&D Systems, Minneapolis, MN, USA) to increase proliferation and treated with Ripasudil (0.3, 3, 30 µM) as a positive control.

In another series of experiments, hRPE were serum starved (medium supplemented with 0.1% FBS) overnight, 24 hours after cell seeding. The medium of the cells was changed to a fresh serum-free medium containing Ripasudil at different concentrations (0.3, 3, 30 µM). Twenty-four hours after growth factor or compound administration, cell proliferation was assessed in all experiments using the MTT Cell Proliferation Assay Kit (Funakoshi Co., Ltd., Tokyo, Japan). A complete or serum-free medium was used as a control.

#### Scratch Assay

The effect on cell migration was evaluated using an in vitro scratch assay, performed as previously described. HhRPE were grown to confluence in 24-well tissue culture dishes and were serum-starved overnight. The next day, the monolayer was scratched with a plastic pipette tip to remove the confluent cells by two perpendicular linear scratches to create linear wounds. The hRPE were incubated at 37°C in a starvation medium. Ripasudil (0.3 3 30 µM) was added to hRPE 24 hours after the stimulation of recombinant human 10 ng/mL TGF-β2. After 24 hours, the movement of cells into the wound area was measured using a crossline micrometer on a Primo Vert 1 microscope (both from Carl Zeiss Meditec). The shortest distance between the edges of migrated cells (including protrusions) from both sides was measured to determine the effect of Ripasudil after wounding a monolayer of cells. These measurements were at four predefined points at the start and 24 hours after wounding.

#### Electroretinography

Scotopic ERGs were obtained using a Micron III retinal imaging system with a focal ERG attachment (Phoenix-MICRON, Inc., Bend, OR, USA). The corneal electrode (gold) was attached to the focal ERG lens mount, whereas the reference and ground electrodes (platinum) were inserted into the mouse and the tail, respectively. Mice were dark-adapted overnight, and the procedure was carried out under low-level red light. The corneal electrode (gold) was attached to the focal ERG lens mount, whereas the reference and ground electrodes (platinum) were inserted the mouse and attached the tail respectively. Mice were exposed to three flashes of white light at 6.8 log cd • s/m^2^ intensity for 1 ms, with each flash separated by 120 seconds to restore dark adaptation. Recordings were obtained for 50 ms pre, and 250 ms post-stimulus using LabScribe ERG v3 software (iWorx, Dover, NH, USA) with a 2–1000 Hz band pass filter, 5 kHz sampling frequency, and 50 Hz noise reduction. An average of three readings were obtained per eye at each time point. The b-wave amplitudes for each retina were calculated to assess photoreceptor and inner retinal function.

#### Immunohistochemical Analysis of Human Ocular Tissue

Human eye samples with fibrosis in nAMD were fixed in 10% neutral buffered formalin, dehydrated, and embedded in paraffin. The deparaffinized sections were incubated in 10 mM citrate buffer (pH 6.0) for one hour at boiling temperature before blocking with 1% BSA in TBS for 30 minutes. The sections were immunolabeled using type1 collagen (35429; Rockland; 1:100), ROCK1 (sc-17794; Santa Cruz; 1:200), ROCK2 (sc-1851; Santa Cruz; 1:200), αSMA (ab21027; Abcam; 1:200) overnight at 4°C. The immuno-binding antibodies were detected using biotin-conjugated secondary antibodies.

#### Statistics

All statistical analyses were performed using graphing software (Prism 8; GraphPad Software). All data are expressed as mean ± SEM. Statistical significance was determined by unpaired two-tailed Student’s *t*-test or ANOVA, followed by Dunnett’s multiple comparison test. *P* values < 0.05 were considered significant.

##### Study Approval

The Animal Care Committee approved all animal experiments of Kyushu University (A19-0151 and A19-2281). All experimental procedures on the animals were performed according to the ARVO Statement for the Use of Animals in Ophthalmic and Vision Research. The use of human subretinal fibrosis tissues was approved by Institutional Ethics Committee in Kyushu University. The study was conducted according to the principles expressed in the Declaration of Helsinki. All patients in this study provided written informed consent for sample collection and data analyses.

## Results

### Fate Mapping Approach to Explore the Origin of Myofibroblasts in SRF

**Figure 1.**
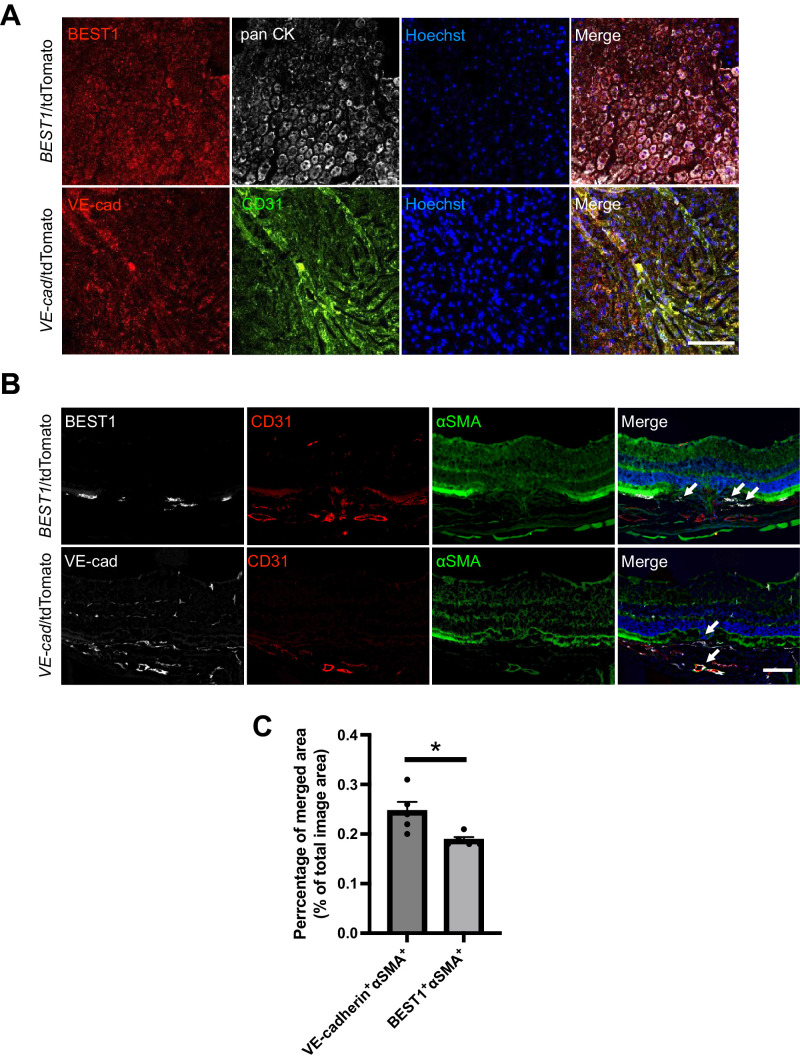
Fate mapping reveals the origin of myofibroblasts in subretinal fibrosis formation. **(A)** Validation of lineage tracing specificity in BEST1-Cre-tdTomato and VE-cadherin-Cre-tdTomato mice by whole-mount immunofluorescence staining. BEST1⁺ cells co-localized with pan-cytokeratin⁺ RPE, and VE-cadherin⁺ cells co-localized with CD31⁺ vascular endothelial cells, confirming the fidelity of tdTomato labeling. **(B)** Lineage tracing of myofibroblasts in a laser-induced CNV model. Immune fluorescent stains confirmed BEST1^+^ or VE-cad^+^ and αSMA^+^ cells co-expression in subretinal fibrosis (activated fibroblasts were expressing αSMA^+^). The allow shows the co-expression part (*Scale bars*: 100 µm). **(C)** Quantitative analysis of immunofluorescence staining showing the percentage of merged area for BEST1⁺/αSMA⁺ and VE-cadherin⁺/αSMA⁺ double-positive cells in subretinal fibrosis. The merged area was calculated as the percentage of total image area. Data were obtained from *n* = 5 samples and are presented as mean ± SEM. Statistical significance was analyzed using an unpaired *t*-test.

To delineate the cellular contribution of EMT and EndMT to SRF, we used the Cre/loxP system to label RPE (BEST1-Cre-tdTomato mice) and vascular endothelium (VE-cadherin-Cre-tdTomato mice). First, we examined the fidelity of tdTomato to adequately label RPE and vascular endothelial cells by whole-mount immunostaining. As predicted, BEST1^+^ cells showed co-staining with pan cytokeratin^+^ cells and VE-cadherin^+^ cells co-staining with CD31^+^ cells, respectively (Fig. 1A). Next, we initiated lineage tracing to investigate the origin of myofibroblasts in SRF. We created SRF in a laser-induced CNV mice model to investigate the overlap between tdTomato^+^ cells and αSMA^+^ cells, a marker of myofibroblasts,^22^ with immunostaining of cryosections of the reporter mice. Within the SRF lesion complex, VE-cadherin^+^ and BEST1^+^ cells co-stained with αSMA^+^ cells (Fig. 1B). Some VE-cadherin^+^ cells were also expressed within the sprouting endothelial cells that form CNV. Quantitative analysis confirmed the presence of both αSMA⁺/VE-cadherin⁺ and αSMA⁺/BEST1⁺ cells, with αSMA⁺/VE-cadherin⁺ cells being significantly more abundant than αSMA⁺/BEST1⁺ cells (0.25% ± 0.04% vs. 0.19% ± 0.01%; *P* < 0.05) (Fig. 1C).These results suggest that the emergence of myofibroblasts within the SRF arises from the RPE and vascular endothelium, wherein both EMT and EndMT processes contribute to this phenomenon.

### Microarray Analysis of SRF With Laser Capture Microdissection

**Figure 2.**
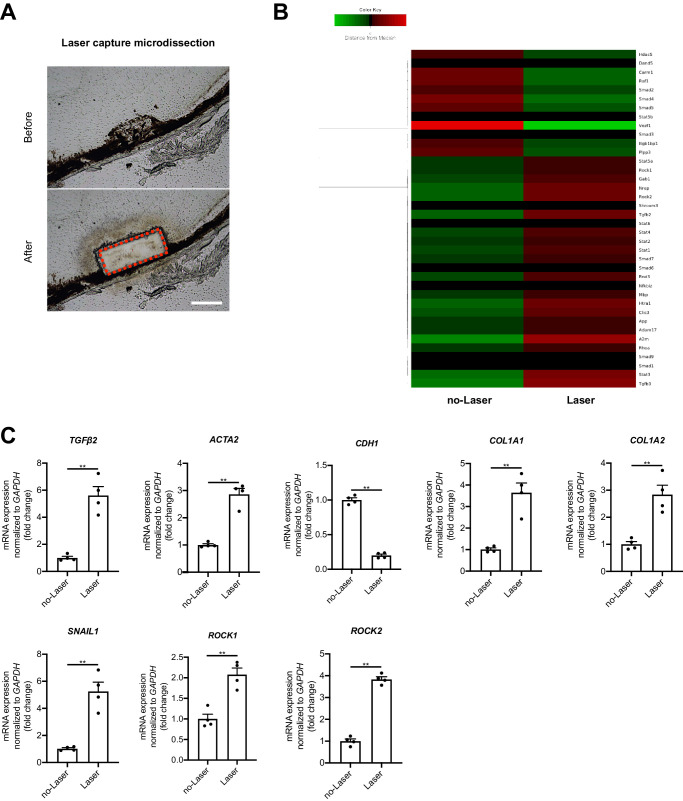
Epithelial-to-mesenchymal transition by TGF-β2–ROCK pathway is involved in subretinal fibrosis formation. **(A)** Histology of laser captured material by LCM (*left*: before, right: after) (*Scale bars*: 100 µm). **(B)** Libraries are subjected to sequencing. Heat maps generated from RNA microarray data reflecting specific fibrosis part expression profiles of the subretinal fibrosis condition 21 days after laser compared with control (in vivo). The listed genes are overexpressed genes (in *red*) in each group. Green corresponds to under-expressed genes. **(C)** Quantitative real-time RT-PCR validation of genes that were differentially displayed in control without fibrosis and specific fibrosis in the laser-induced CNV model by LMD. Values are the means ± SD. *P < 0.05, **P < 0.005, Student's *t*-test.

We excised the specific SRF lesions by using laser capture microdissection (LCM) on day 21 after laser induction to investigate the gene expression patterns (Fig. 2A). Wild-type mice without laser-induction were used as a control group. RNA quality, as well as lack of contamination with other cell types, was confirmed with Bioanalyzer and PCR analyses in the collected samples of SRF lesions. Microarray analysis identified that Rho-related gene expression was elevated in SRF compared to controls (Fig. 2B). The Rho/ROCK signaling pathway is involved in various cellular functions, including cell proliferation, migration, and contraction.^32^ It has been reported to play a crucial role in cardiovascular diseases, central nervous system diseases, and cancer, making it an important therapeutic target for various diseases, including retinal diseases.^33^^–^^38^

Next, we performed real-time RT-PCR on the RNAs extracted from specific-SRF lesions by LCM and compared them with the microarray analysis results to validate them. Similar to the results of microarray analysis, RT-PCR analysis detected significantly higher expression of *ROCK1* and *ROCK2* genes in the SRF group compared to the control group. In addition, this analysis confirmed EMT- and EndMT-related genes, *Tgfβ2, ACTA2* (αSMA), *COL1A1*, *COL1A2*, and *SNAIL1*, in the SRF in comparison to control. In contrast, the gene expression of *CDH1* (E-cadherin) was significantly lower (Fig. 2C). These results indicate that Rho-related genes are involved in SRF and could correlate with mesenchymal transition through activating TGF-β2 signaling.

### ROCK Signaling Contributes to SRF Through EMT in RPE

**Figure 3.**
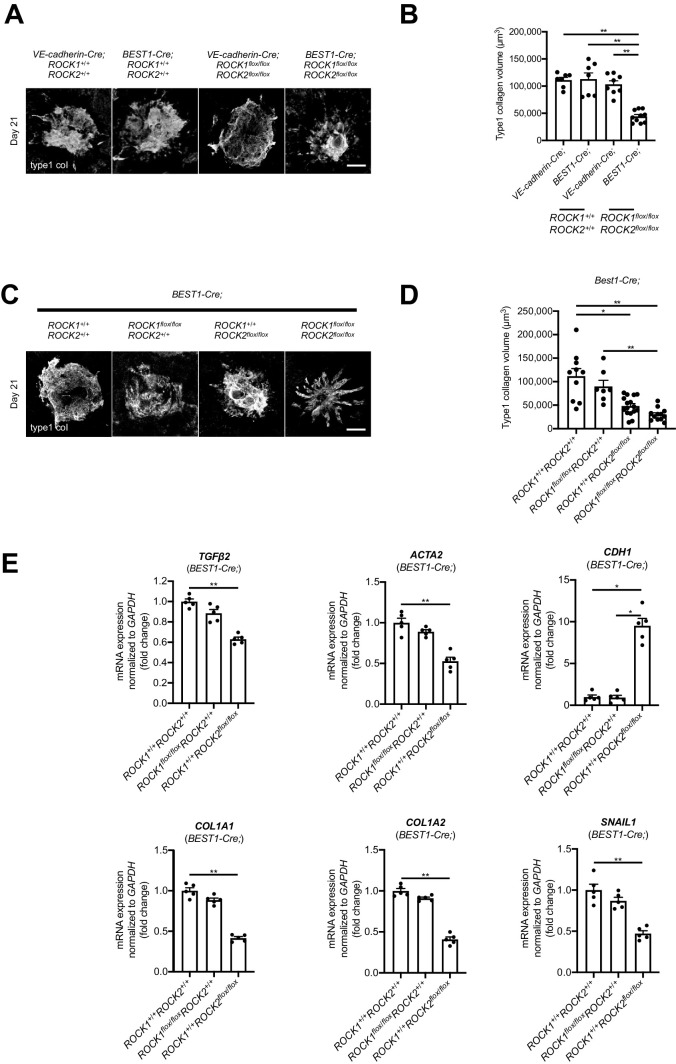
TGF-β2–ROCK pathway in RPE cells is involved in subretinal fibrosis formation. **(A,** **B)** Immune fluorescent stains confirmed whether SRF (type1 collagen^+^ cell) was reduced in RPE cell-specific (*BEST1^Cre^*) or vascular endothelial cell-specific (VE-cad*^Cre^)* ROCK 1 or 2 KO mice (*Scale bars*: 100 µm). Type1 col: type1 collagen. Values are the means ± SD. *P < 0.05, **P < 0.005, Student's *t*-test. *n* = 7–10 laser spots. **(C,** **D)** Immune fluorescent stains confirmed whether SRF (type1 collagen^+^ cell) was reduced in RPE cell-specific ROCK1 KO (*BEST1^Cre^*; ROCK1*^flox/flox^*ROCK2*^+/+^*), ROCK2 KO (*BEST1^Cre^*; ROCK1*^+/+^*ROCK2*^flox/flox^*), and ROCK1 and 2 KO (*BEST1^Cre^*; ROCK1*^flox/flox^* ROCK2*^flox/flox^*) mice (*Scale bars*: 100 µm). Values are the means ± SD. *P < 0.05, **P < 0.005, Student's *t*-test. *n* = 7–16 laser spots. **(E)** Expressions of the mRNAs of various cytokines related to EMT and MET in RPE cell-specific ROCK1 KO (*BEST1^Cre^*; ROCK1*^flox/flox^*ROCK2*^+/+^*), ROCK2 KO (*BEST1^Cre^*; ROCK1*^+/+^*ROCK2*^flox/flox^*), and ROCK1 and 2 KO (*BEST1^Cre^*; ROCK1*^flox/flox^* ROCK2*^flox/flox^*) mice by LCM. Values are the means ± SD. *P < 0.05, **P < 0.001, Student's *t*-test. *n* = 5 eyes, 20 laser spots.

We generated RPE-specific *ROCK1* or/and *ROCK2*-conditional knockout (cKO) mice and vascular endothelium-specific *ROCK1* or/and *ROCK2*-cKO mice to investigate the relationship between ROCK and EMT/EndMT in the SRF. Fibrotic areas were quantified by type1 collagen staining with laser-induced CNV model, and the type1 collagen^+^ cells were significantly reduced in RPE-specific *ROCK1* and *ROCK2-*cKO mice compared with control and vascular endothelium-specific mice (*P* < 0.001). Interestingly, there is no significance in vascular endothelium-specific *ROCK1* and *ROCK2-*cKO mice compared with control (Figs. 3A, 3B). Thus ROCK signaling in RPE cells appears to be the principal contributor to SRF formation, yet the involvement of endothelial-derived processes is not completely excluded.

Next, we generated two types of transgenic mice, RPE-specific *ROCK1-*cKO mice and *ROCK2-*cKO mice*,* to investigate which isoform, ROCK1 or ROCK2, was regulated SRF formation in RPE cells. The type1 collagen^+^ cells were significantly suppressed in RPE-specific *ROCK2-*cKO mice compared with control mice (*P* < 0.05), although there is no significance in RPE-specific *ROCK1-*cKO mice. Moreover, type1 collagen^+^ cells were significantly suppressed in RPE-specific *ROCK1* and *ROCK2-*cKO mice compared with *ROCK1-*cKO mice (*P* < 0.05) (Figs. 3C, 3D). These results indicate that ROCK2 is more involved in SRF formation in RPE cells than ROCK1.

Finally, we investigated whether ROCK signaling in RPE cells is involved in EMT by RT-PCR. Samples were collected specifically in fibrotic areas by LCM. EMT-associated genes *ACTA2* (αSMA) and *SNAIL1* were significantly suppressed (*P* < 0.001), whereas the epithelium-associated gene, *CDH1* (E-cadherin), was significantly upregulated in RPE-specific *ROCK2-*cKO mice (*P* < 0.001), not in RPE-specific *ROCK1* mice. Moreover, *TGF-β2* was significantly suppressed in RPE-specific *ROCK2-*cKO mice compared to control and RPE-specific *ROCK1-*cKO mice (*P* < 0.001) (Fig. 3E). These results suggest that ROCK signaling–mediated EMT plays an important role in SRF formation in RPE cells and may also participate in a reciprocal regulatory loop with TGFβ2 signaling upstream of ROCK.

### ROCK Inhibitors Suppress SRF Formation in Laser-Induced CNV Model in Mice

**Figure 4.**
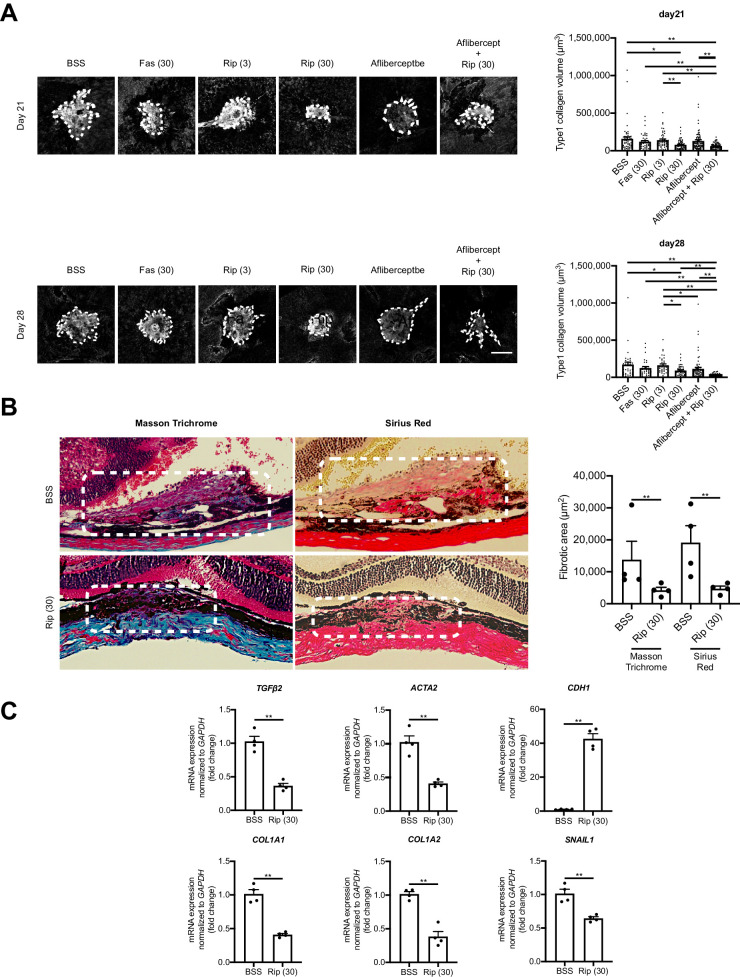
ROCK inhibitor suppresses subretinal fibrosis. **(A)** Immune fluorescent stains confirmed whether SRF (type1 collagen^+^ cell) was reduced by intravitreal (IVT) injections of BSS as control, Fasudil (30 µM), which was another ROCK inhibitor, Ripasudil (3, 30 µM), and Ripasudil (30 µM) with Aflibercept (400 ng/mL), which was anti-VEGF therapy (*Scale bars*: 100 µm). Fas 30, Fasudil 30 µM; Rip 3, Ripasudil 3 µM; Rip 30, Ripasudil 30 µM; Afli, Aflibercept. Values are the means ± SD. *P < 0.05, **P < 0.005, Student's *t*-test. *n* = 20 mice. **(B)** Masson's trichrome and Sirius red stains confirmed whether SRF (type1 collagen^+^ cell) was reduced by IVT injections of control and Ripasudil (30 µM) Values are the means ± SD. *P < 0.05, **P < 0.005, Student's *t*-test. *n* = 10 mice. **(C)** Expressions of the mRNAs of various cytokines related to EMT and MET in SRF-specific part by laser capture microdissection (LCM) after IVT injections of control or Ripasudil (30 µM). Values are the means ± SD. *P < 0.05, **P < 0.01, Student's *t*-test**.** *n* = 5.

Next, we examined the effect of Ripasudil, a selective inhibitor for ROCK1 and ROCK2, on SRF in the laser-induced CNV model in mice. The SRF volume was quantified using confocal microscopy on whole-mounts stained with type 1 collagen on days 21 and 28 after laser injury. To minimize the influence of CNV-related EndMT on SRF, all treatments were administered 14 days after laser induction, because CNV regression has been observed at the time point.^39^ Intravitreal injections were repeated on days 14, 17, 20, 23, and 26, including BSS as a control, Fasudil (30 µM; another ROCK inhibitor), Ripasudil (3, 30 µM), and Ripasudil (30 µM) with Aflibercept (anti-VEGF therapy). Treatment with high-concentration Ripasudil (30 µM) significantly decreased type1 collagen^+^ cell volumes on both days (43.2% and 46.6% reduction from baseline at 30 µM Ripasudil; *P* < 0.01 and *P* < 0.05). Fasudil and Aflibercept treatment also reduced the volume of type1 collagen^+^ cells compared to the control group but not as robustly or significantly as Ripasudil. Moreover, Ripasudil with Aflibercept significantly reduced the volumes of type I collagen+ cells (*P* < 0.01); nevertheless, no significant difference was observed when compared with Ripasudil monotherapy (Fig. 4A). Masson trichrome and Sirius red staining also showed that the Ripasudil (30 µM) treatment significantly reduced SRF (*P* < 0.05 and *P* < 0.05) (Fig. 4B). These results show that Ripasudil could be a candidate therapeutic for SRF independently of Aflibercept, while also suggesting that Ripasudil may retain similar efficacy even when administered alongside Aflibercept.

Next, we performed RT-PCR analysis to investigate the effect of Ripasudil with EMT on specific SRF areas by using LCM. The results showed that Ripasudil (30 µM) significantly decreased mRNA expression of EMT-related genes (*TGF-β2*, *αSMA*, *COL1A1*, *COL1A2*, and *SNAIL1*) compared with the control. In contrast, the epithelium-related gene, *CDH1*, was significantly increased by Ripasudil (30 µM) (*P* < 0.005) (Fig. 4C). These results indicate that ROCK inhibitor Ripasudil decreases the SRF via the suppression of EMT-related gene expression in vivo model.

### ROCK Inhibitors Normalize Human RPE via the Regulation of EMT/MET

**Figure 5.**
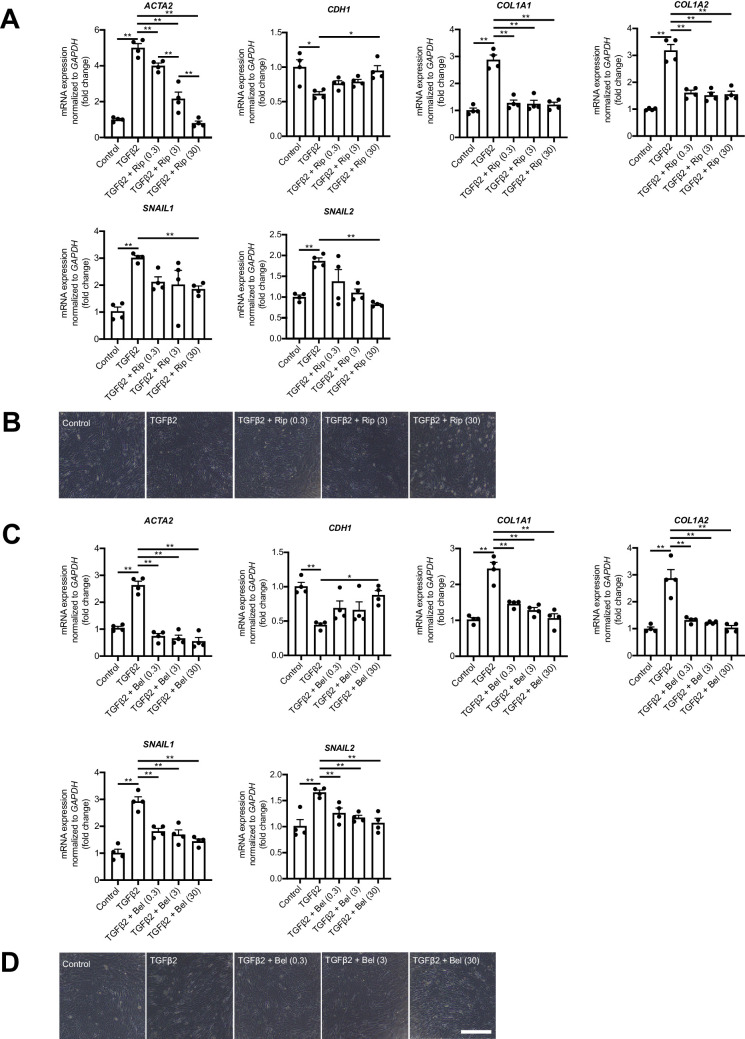
ROCK inhibitor and ROCK2 selective inhibitor normalize hRPE cell through EMT suppression and MET enhancement. **(A, C)** a: Ripasudil, c: belumosudil. Expressions of the mRNAs of various cytokines related to EMT and MET in the hRPE after Ripasudil (0.3, 3, 30 µM) adding with TGF-β2 (10 ng/mL) stimulation. **(B,** **D)** Representative pictures of the shape of hRPE with control, TGF-β2 (10 ng/mL), or Ripasudil (0.3, 3, 30 µM) after TGF-β2 (10 ng/mL) stimulation**.** (*Scale bars*: 200 µm) Rip 0.3, Ripasudil 0.3 µM; Rip 3, Ripasudil 3 µM; Rip 30, Ripasudil 30 µM; Bel 0.3, Belumosudil 0.3 µM; Bel 3, Belumosudil 3 µM; Bel 30, Belumosudil 30 µM. Values are the means ± SD. *P < 0.05, **P < 0.001, Student's *t*-test**.** *n* = 5.

We performed RT-PCR analysis with human RPE (hRPE) cells to investigate the effect of Ripasudil in vitro. The results showed that Ripasudil (0.3, 3, 30 µM) significantly suppressed the upregulation of EMT-related genes (*αSMA*, *COL1A1*, *COL1A2*, *SNAIL1*, and *SNAIL2*) by TGF-β2 (10 ng/mL) treatment. Conversely, the epithelium-related gene (*CDH1*), which was significantly suppressed by TGF-β2 treatment (*P* < 0.005), was significantly increased by Ripasudil (3 and 30 µM) (*P* < 0.05) (Fig. 5A). Interestingly, Ripasudil reversed TGF-β2-introduced spindle-shaped hRPE cells to regular hexagonal structure (Fig. 5B). In vivo studies revealed higher involvement of ROCK2 in SRF. Therefore we explored the impact of the ROCK2-specific inhibitor, Belumosudil, on EMT-related genes in vitro RPE cells (Figs. 5C, 5D). Belumosudil (0.3, 3, 30 µM) also significantly suppressed the TGF-β2 (10 ng/mL)-induced upregulation of EMT-related genes (α*SMA*, *COL1A1*, *COL1A2*, *SNAIL1*, and *SNAIL2*) (*P* < 0.005) and increased the expression of the epithelium-related gene (*CDH1*), which was significantly suppressed following TGF-β2 treatment (*P* < 0.05) (Fig. 5C). Belumosudil also reversed TGF-β2-introduced spindle–shaped hRPE cells to regular hexagonal structure (Fig. 5D).

Next, time-lapse imaging system confirmed the real-time changes of hRPE cells by Ripasudil from spindle-shape. The hRPE cells, induced into a fibroblast-esque morphology following a 24-hour stimulation of TGF-β2 (Supplementary Video S1A), exhibited a restoration towards a morphology to that of normal epithelial cells upon the subsequent introduction of Ripasudil (Supplementary Video S1B). These results also showed that Ripasudil normalized hRPE cells once mesenchymalized by TGF-β2 treatment.

### ROCK Inhibitors Enhance Epithelial Expression in Normal Human RPE Cells

**Figure 6.**
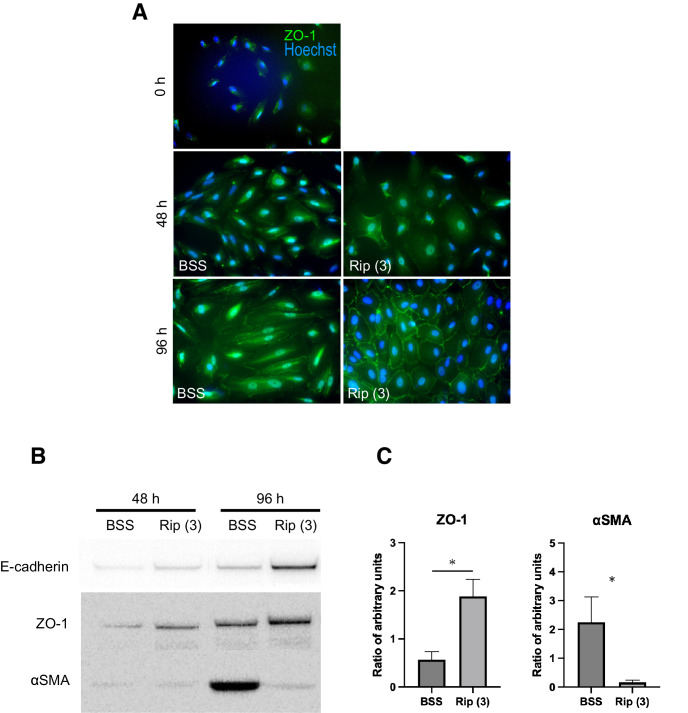
Ripasudil normalizes hRPE through cell-to-cell adhesion. **(A)** Immunofluorescence staining for ZO-1 (*green*) was performed to assess cell-to-cell adhesion in hRPE cells treated with BSS as control or Ripasudil (3 µM). At the 0-hour time point, ZO-1 expression was minimal, and spindle-shaped cells were rarely observed. After incubation with culture medium, hRPE cells adopted a spindle-like morphology, and ZO-1 expression at the cell periphery became discontinous. Treatment of Ripasudil reversed the morphology of hRPE cells to cobblestone-like arrangement and enhanced the ZO-1 expression. **(B)** Western blot analysis of E-cadherin, ZO-1, and αSMA in the cell lysates of RPE. Rip 3, Ripasudil 3 µM. **(C)** Densitometric quantification of Western blot bands for ZO-1 and αSMA normalized to β-actin or GAPDH after 96 hours of treatment. Relative protein expression levels are presented as ratios to BSS-treated controls. Ripasudil (3 µM) significantly increased ZO-1 expression and decreased αSMA expression compared with BSS. Data are shown as mean ± SD from three independent experiments (*n* = 3). *P < 0.05 versus BSS (Student's *t*-test).

We next investigated the impact of Ripasudil on ZO-1 expression in hRPE cells. After the incubation with medium, hRPE cells changed to spindle-like shapes and ZO-1 expression at the cell periphery became intermittent. Ripasudil reversed the morphology of hRPE cells to cobblestone-like arrangement and enhanced the ZO-1 expression (Fig. 6A). Western blotting analysis also confirmed that the upregulation of ZO-1 and E-cadherin expressions by Ripasudil treatment, whereas αSMA was suppressed (Figs. 6B, 6C). These results suggest that Ripasudil could normalize hRPE cells by improving cell-cell adhesion, an important observation given the important role of the RPE in maintaining homeostasis.

### ROCK Inhibitors Inhibit the Migration and Proliferation of Human RPE Cells

Migration and proliferation of mesenchymalized cells are known to be essential steps in the fibrosis development.^40^ Therefore we examined the effects of Ripasudil on migration and proliferation in hRPE cells. High-concentration Ripasudil (30 µM) significantly suppressed cell migration and proliferation under TGF-β2 (10 ng/mL) treatment in a dose-dependent manner both (*P* < 0.001 and *P* < 0.05, respectively) (Supplementary Figs. S1A, S1B). These results indicated that Ripasudil has the potential to inhibit of the migration, as well as proliferation in hRPE cells, which can contribute to the SRF formation.

### ROCK Inhibition Target Both Angiogenesis and Fibrosis Whereas Anti-VEGF Agents Cannot Inhibit Fibrosis

**Figure 7.**
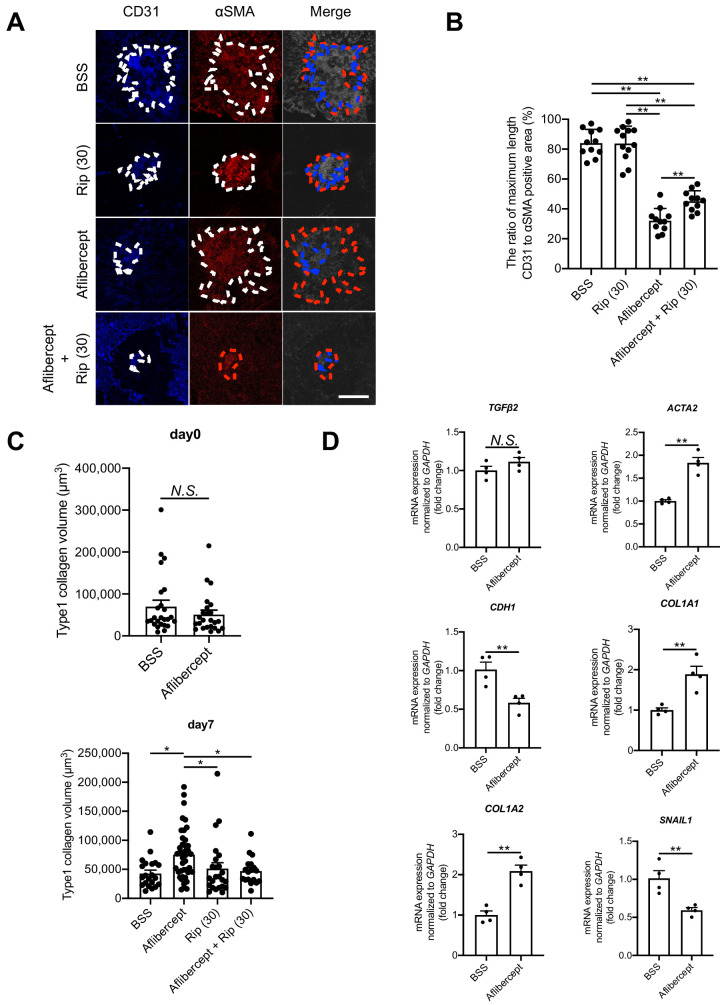
ROCK inhibitor control angio-fibrotic switch in subretinal fibrosis via epithelial-to-mesenchymal transition suppression. **(A,** **B)** Immune fluorescent stains compared between vascular component (CD31^+^, *blue*) and SRF (activated fibroblasts expressing αSMA^+^, *red*). They were performed by IVT injections of BSS as control, Ripasudil (30 µM), and Ripasudil (30 µM) with Aflibercept (400 ng/mL) (*Scale bars*: 100 µm). *n* = 20 mice. **(C)** Immune fluorescent stains confirmed whether SRF (type1 collagen^+^ cell) was reduced by IVT injections of BSS as control, Ripasudil (30 µM), and Ripasudil (30 µM) with Aflibercept (400 ng/mL), which was anti-VEGF therapy. *n* = 20 mice. **(D)** Expressions of the mRNAs of various cytokines related to EMT and MET in SRF-specific part by LCM after IVT injections of control or Aflibercept (400 ng/mL). *n* = 5. Rip 30, Ripasudil 30 µM. Values are the means ± SD. *P < 0.05, **P < 0.005, Student's *t*-test.

We investigated the effect of anti-VEGF therapy on SRF in the laser-induced CNV mouse model. Aflibercept significantly reduced the area of CD31^+^ cells but not αSMA^+^ cells, whereas the combination treatment with Aflibercept and Ripasudil significantly reduced both areas of CD31^+^ and αSMA^+^ cells (27.1% reduction from baseline at Ripasudil; *P* < 0.01 and 22.9% reduction from baseline at the combination treatment; *P* < 0.05) (Figs. 7A, 7B). This result implicated that could specifically inhibit angiogenesis but not SRF, whereas Ripasudil could impact angiogenesis, as well as SRF.

The controversy regarding the suppressive effects of anti-VEGF therapy on fibrosis persists,^9^^,^^41^ with studies suggesting both suppression and augmentation. In the context of proliferative diabetic retinopathy, anti-VEGF injections have been associated with increased fibrosis due to an imbalance between connective tissue growth factor and VEGF.^42^ Therefore we investigated the time-dependent impact of anti-VEGF therapy on SRF during CNV formation in a mouse model. It has been reported that CNV volume is the largest at day 7 whereas SRF continues to develop in the CNV model even beyond this time point.^39^ Aflibercept was administered every three days for two weeks, starting either from day 0 or day 7. The group of treated with Aflibercept from day 7 showed a significantly greater volume of type1 collagen^+^ cells compared to the control (*P* < 0.05). In contrast, combination therapy with Aflibercept and Ripasudil (from day 7) significantly inhibited the acceleration of type1 collagen^+^ volume (*P* < 0.05) (Fig. 7C), suggesting that Ripasudil could prevent accelerated SRF development induced by anti-VEGF therapy.

Moreover, to analyze how anti-VEGF therapy affects the development of SRF, RT-PCR analysis with LCM was performed in this model. Interestingly, *TGF-β2* was not significantly upregulated in the anti-VEGF therapy group compared to the control group. In contrast, EMT-related genes *ACTA2* (αSMA), *COL1A1*, *COL1A2*, and *SNAIL1* were significantly upregulated (*P* < 0.05), and epithelial-related gene *CDH1* (E-cadherin) was significantly decreased with anti-VEGF therapy compared to the control group (*p* < 0.05) (Fig. 7D). These results suggest that another EMT pathway, not mediated by TGF-β signaling, involved in the fibrosis-promoting mechanism of anti-VEGF therapy.

### ROCK Inhibitor Improves Retinal Function in Laser-Induced CNV Model in Mice

**Figure 8.**
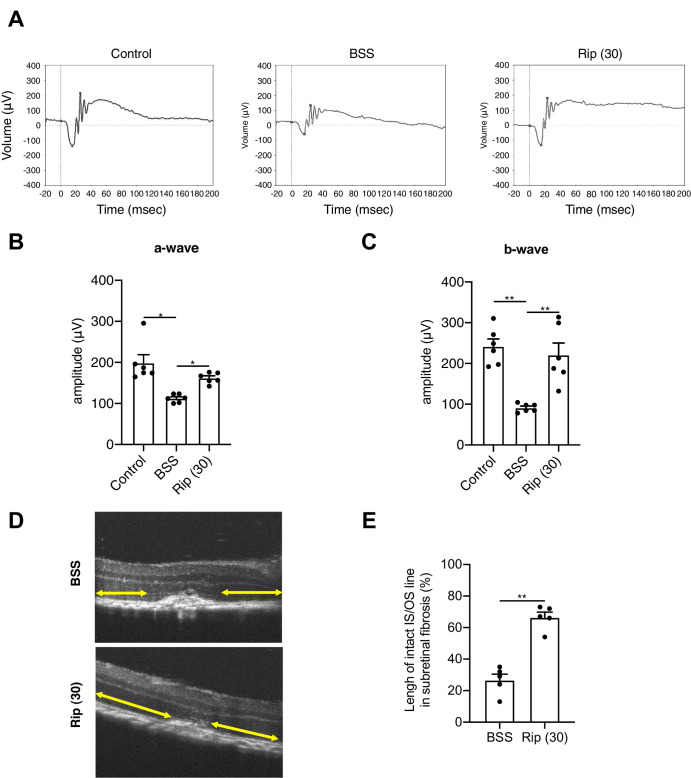
ROCK inhibitor clinically improved retinal function. **(A)** Electroretinogram (ERG) analysis of rod and cone function in laser-induced choroidal neovascularization (CNV) model mice. The a-wave represents the hyperpolarization of photoreceptors, and the b-wave represents the second-order neuron response. **(B, C)** No laser group as control. Intravitreal (IVT) injections performed balanced salt solution (BSS), Ripasudil (30 µM) in laser group. **(D, E)** The junction between the photoreceptor inner and outer segment (IS/OS) line lengths in spectral domain optical coherence tomography (SD-OCT). These lines were measured in the foveal center. The yellow arrow represents the IS/OS line. IVT injections performed balanced salt solution (BSS) as control, Ripasudil (30 µM) in laser group. BSS, balanced salt solution; Rip 30, Ripasudil 30 µM; Afli, Aflibercept. Values are the means ± SD. *P < 0.05, **P < 0.005, Student's *t*-test. *n* = 5.

Next, we investigated the clinical implications of ROCK inhibitors for SRF. Early AMD patients are known to show a reduction in a-wave (photoreceptor rods/cones) and b-wave (inner retina, predominantly Müller and ON-bipolar cells) amplitudes.^43^ Consistent with clinical observations, both a- and b-wave significantly decreased after the twenty-first day after 20 shots laser-induction compared to control (without laser induction) in mice (*P* < 0.001). However, Ripasudil significantly improved both waves (*P* < 0.05) (Figs. 8A–C). Furthermore, retinal imaging by optical coherence tomography revealed that Ripasudil also prevented laser-induced alteration of the junction between the photoreceptor inner and outer segment (IS/OS) line (ellipsoid zone, EZ line) (*P* < 0.001) (Figs. 8D, 8E). These results showed that Ripasudil could also protect photoreceptor and the neural retina by suppression of SRF.

### ROCK Expression in Human AMD Samples

Finally, we investigated ROCK expression by immunohistochemistry in human samples which choroidal neovascular membranes (CNVMs) harvested from cases with nAMD patients. This sample was from a 78-year-old male with type 1 CNV, which had been surgically removed and stored in paraffin sections. CNVMs samples showed increased expression of αSMA and decreased expression of ZO-1 as the in vivo model. The membrane surface of the sample sections exhibited robust expression of type 1 collagen^+^ cells. ROCK1^+^ and ROCK2^+^ cells, on the other hand, demonstrated expression surrounding the cell nucleus and filling the cell stroma. Importantly, a subset of ROCK2^+^ cells showed co-staining with type 1 collagen^+^ cells on the membrane (Supplementary Fig. S2A). We also found that both ROCK1 and ROCK2 were expressed in some αSMA^+^ cells (Supplementary Fig. S2B).

## Discussion

Myofibroblasts are key components in SRF formation despite the lack of fibrocytes in the macula during homeostatic conditions.^44^ Previous histological and molecular studies from AMD patients and SRF animal models have attempted to find the cellular source and have suggested the several candidates including RPE (EMT), vascular endothelium (EndMT), and macrophage.^22^^,^^24^^,^^45^^–^^47^ In this study, we used microarray analysis with LCM and confirmed that SRF activation mediated by the TGF–ROCK axis was driven by EMT rather than by EndMT. A widely-used animal model using cell-specific ROCK isoform knockout mice showed EMT involvement and ROCK2 as the molecular in the SRF formation.^48^ Next, for clinical application, we confirmed a significant suppression effect of SRF via EMT inhibition using clinically approved ROCK inhibitors. In vitro studies using ROCK inhibitors similarly showed EMT suppression of RPE, but interestingly, ROCK inhibition was shown not only to suppress EMT but also to promote MET. ROCK inhibitors have been reported to suppress EMT in another organ disease.^49^^,^^50^ Previous studies have shown that ROCK inhibition reduces SRF in in vivo and in vitro models. For instance, AMA0428, a ROCK inhibitor, attenuated SRF formation in a laser-induced CNV model.^51^ Another ROCK inhibitor, Y-27632, has been reported to induce elongation of RPE cells and reorganization of cytoskeletal microfilaments and microtubules.^52^ Importantly, our current study newly demonstrates that ROCK inhibition suppresses SRF even in a condition where VEGF inhibition fails to prevent SRF formation—an observation that mirrors clinical situations in patients with MNV secondary to AMD undergoing anti-VEGF therapy. Finally, we observed that ROCK inhibitors were safe after intraocular injection and led to recovery of visual function on electroretinography in addition to the therapeutic effects on resolution of SRF. The enzyme inhibitory effect of Ripasudil used in this study is approximately five to 10 times stronger than that of conventional ROCK inhibitors, such as Fasudil.^53^ ROCK expression in SRF was also confirmed in patient samples. Taken together, these results collectively indicated that we identified the cellar origin of myofibroblasts in SRF and elucidated their molecular mechanisms and their therapeutic utility.

Histological analysis has pointed out that CNVMs are composed of connective tissue such as ECM and various cellular components such as vascular endothelial cells, RPE cells, macrophages, myofibroblasts, pericytes, fibroblast-like cells.^44^^,^^54^ Previous studies using the same mouse model we used in this study reported pericytes and macrophages as candidates for causing fibrosis.^24^ However, in this study, we focused on vascular endothelial cells and RPE cells in SRF and confirmed their involvement. Taken together, it is possible that a variety of cells are involved in the process.

Immunohistochemistry analyses demonstrated the presence of αSMA⁺/VE-cadherin⁺ cells and αSMA⁺/BEST-1⁺ cells, suggesting that both EndMT and EMT may contribute to SRF formation in this study. Subsequent analyses using tissue-specific ROCK conditional knockout mice revealed that the Rho/ROCK signaling pathway affects EMT in RPE cells more strongly than EndMT in endothelial cells. Thus, although both EMT and EndMT contribute to SRF formation, these results indicate that ROCK-dependent regulation is particularly relevant to EMT in RPE cells.

In addition, the laser-induced CNV mouse model is known to undergo spontaneous regression over time.^39^ To minimize the influence of CNV regression, we initiated our analysis from day 21 after laser induction. Future investigations using alternative time points or different disease models will be necessary to further clarify the temporal dynamics of EndMT and its interplay with EMT in SRF formation.

This study found that the volume of type1 collagen^+^ cells was significantly suppressed in *BEST1-Cre; ROCK1^+/+^ROCK2^flox/flox^* mice compared with both control (wild-type) and *BEST1-Cre; ROCK1^flox/flox^ROCK2^+/+^* mice. Moreover, the volume was most significantly suppressed in *BEST1-Cre; ROCK1^flox/flox^ROCK2^flox/flox^*. The different roles of ROCK1 and ROCK2 has been reported in various diseases.^16^ The previous study showed that ROCK1- and ROCK2-haploinsufficient mice exhibited similar protection from bleomycin-induced vascular leak, myofibroblast differentiation, and fibrosis in pulmonary. However, ROCK1-haploinsufficient mice involved the epithelial cell apoptosis compared with ROCK2-haploinsufficient mice, whereas ROCK2 regulates TGF-β-induced expression of CTGF and profibrotic genes via NF-κB and cytoskeleton dynamics in mesangial cells.^55^ Regulation of the fibrosis mechanism by ROCK2 has also been reported in diabetic kidney disease.^56^^,^^57^ ROCK1 is involved in apoptosis in renal diseases via mitochondrial dysfunction and impaired fatty acid metabolism in glomerular epithelial cells. At the same time, ROCK2 has been reported to regulate fibrosis via the Notch signaling pathway.^58^ We also previously showed the contribution of ROCK2 in choroidal neovascularization in AMD.^16^ Although the function of ROCK1 and ROCK2 in SRF could also be different, as in other fibrotic diseases, our current data showed ROCK2 could play a more critical role in SRF.

In in vitro experiments, both the pan-ROCK inhibitor Ripasudil and the ROCK2-selective inhibitor Belumosudil significantly suppressed EMT-related gene expression in RPE cells. However, these two compounds were not directly compared under the same experimental conditions. Therefore the apparent difference in efficacy between Ripasudil and Belumosudil does not indicate an inconsistency with the genetic findings. Rather, the significant EMT suppression observed with Belumosudil alone supports that selective inhibition of ROCK2 is sufficient to modulate EMT in RPE cells. This finding further strengthens the interpretation that ROCK2 plays a key regulatory role in ROCK-dependent fibrotic signaling, while not excluding a potential minor contribution from ROCK1.

Similar to the current experimental results, the potential role of ROCK in regeneration has been discussed in other ophthalmic diseases.^59^ For instance, in bullous keratopathy, the use of ROCK inhibitors has been shown to promote maximum proliferation of human corneal endothelial cells.^60^ While the precise mechanisms underlying these effects remain unknown, these observations suggest that ROCK may be involved in cell protection and dedifferentiation processes.

This study has several limitations. EMT and EndMT were primarily evaluated using fate mapping and immunohistochemical analyses, and quantitative single-cell validation was limited. Future studies using complementary approaches, including flow cytometry or single-cell transcriptomic analysis, would further strengthen the understanding of the cellular origins of myofibroblasts in SRF.

## Conclusions

The formation of SRF involves the migration of RPE and vascular endothelium to mesenchymal cells, and ROCK signaling may play an important role, involving especially in RPE. Furthermore, the ROCK inhibitor, Ripasudil, not only inhibits but may also repair SRF lesions in AMD. Ripasudil is already approved for ophthalmic use in Japan, and its established safety profile may facilitate future clinical translation.^61^ It may become a novel therapeutic strategy for SRF in AMD, for which no treatment has been available.

## Supplementary Material

Supplement 1

Supplement 2

Supplement 3
