# Supplementary material for: RPE With ROCK-Mediated Epithelial-Mesenchymal Transition as a Key Contributor of Subretinal Fibrosis in AMD
Source: Invest Ophthalmol Vis Sci. 2026 May 14;67(5):33. doi: 10.1167/iovs.67.5.33 (PMC13189205; doi:10.1167/iovs.67.5.33)
Supplement: Supplement 1 [file iovs-67-5-33_s001.pdf]

A

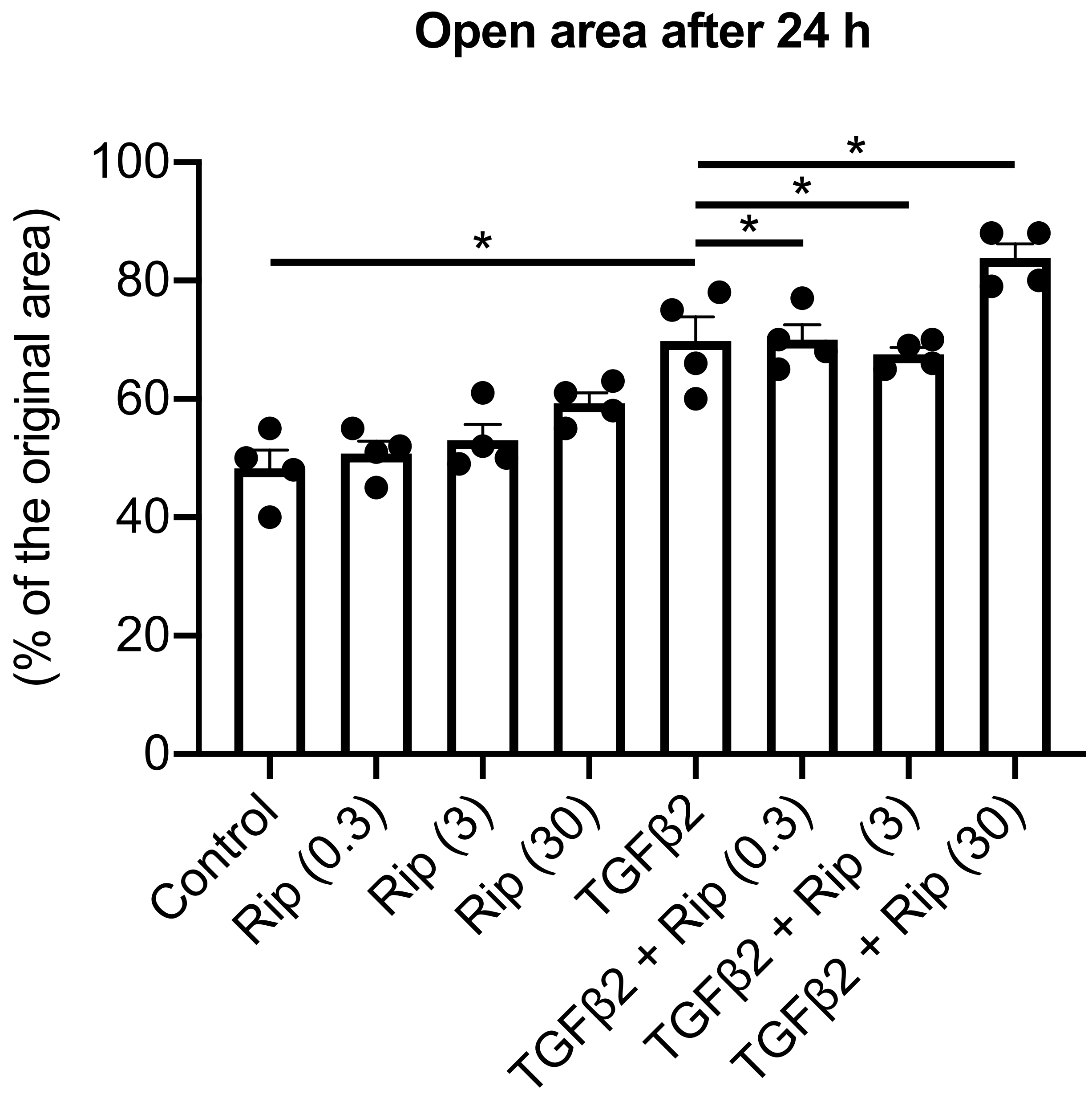

B

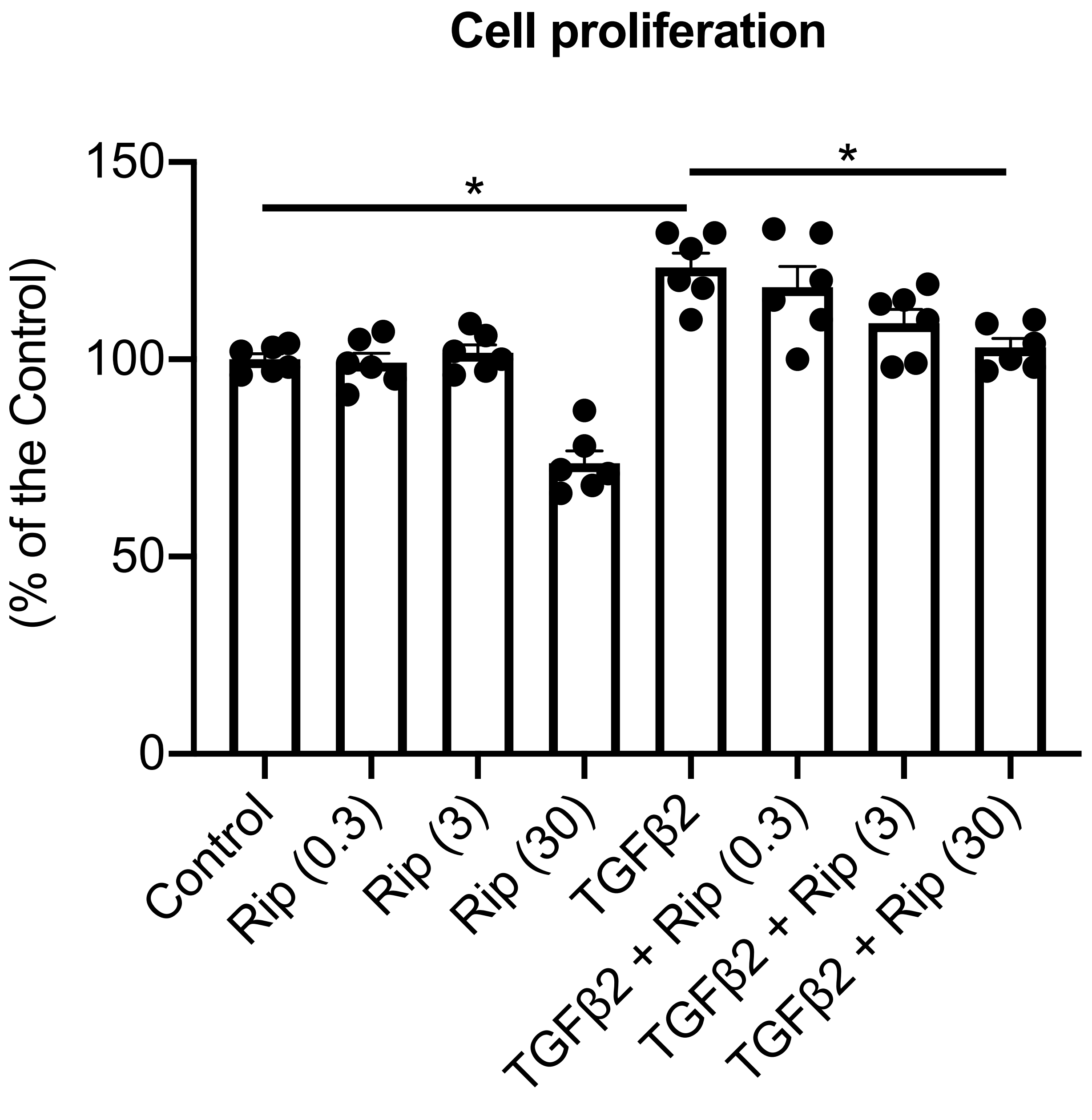

Supplementary Figure 1

Supplementary Figure 1. Effects of ROCK inhibition on migration and proliferation of human RPE cells. (*A*) Quantification of wound closure in hRPE cells treated with ripasudil (0.3, 3, or 30 μM), TGFβ2 (10 ng/mL), or TGFβ2 in combination with ripasudil. The percentage of open area remaining at 24 hours after scratch injury is shown. High-dose ripasudil (30 μM) significantly suppressed TGFβ2-induced cell migration in a dose-dependent manner. (*B*) Cell proliferation of hRPE cells under the same treatment conditions. Proliferation rates were normalized to control. Ripasudil (30 μM) significantly reduced TGFβ2-induced proliferation. Values are the means ± SEM. Rip: ripasudil *$p < 0.05$, **$p < 0.001$. n=4.

Supplementary Figure 1

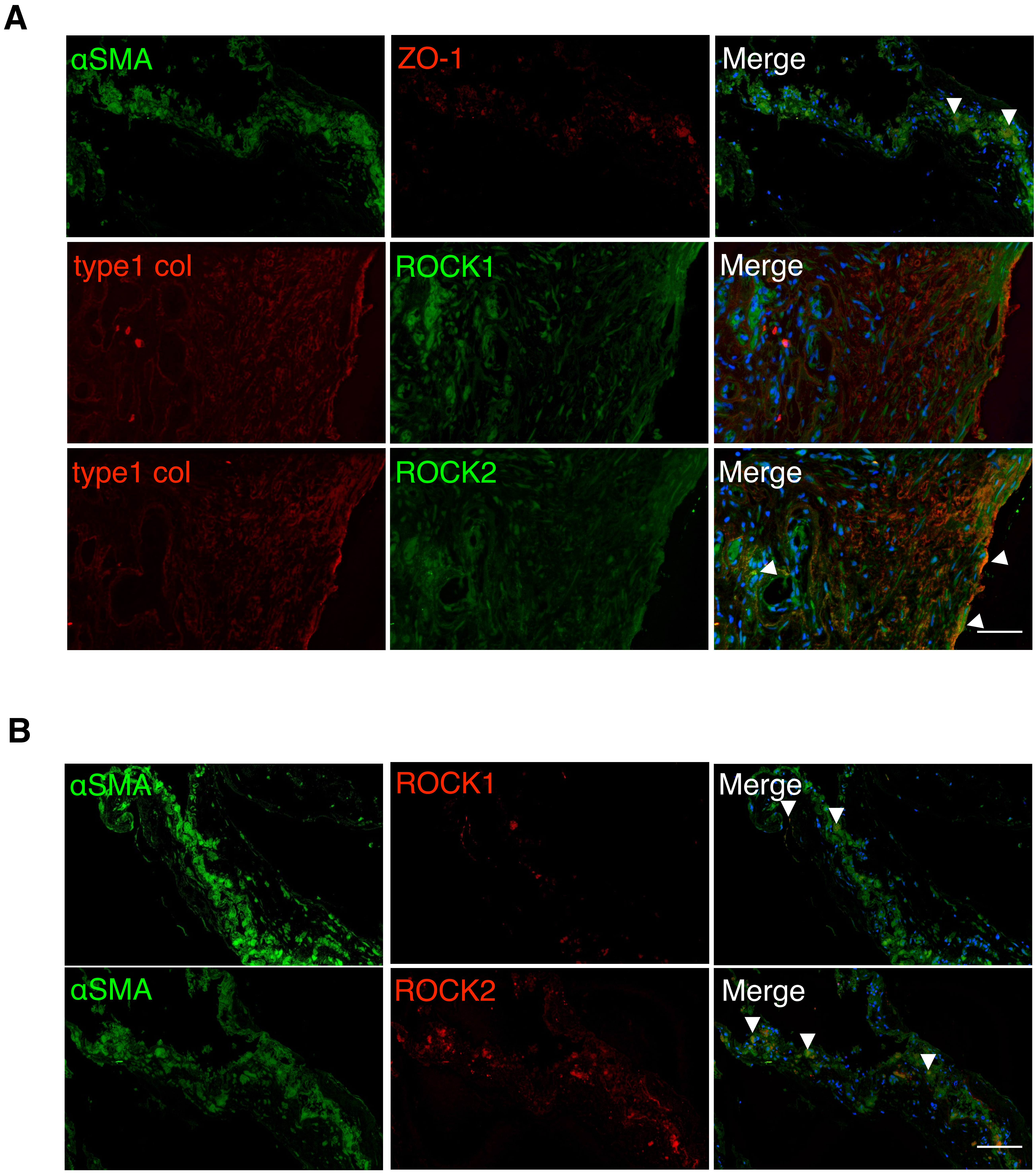

Supplementary Figure 2

Supplementary Figure 2. ROCK expression in choroidal neovascular membranes from a patient with neovascular AMD. (*A*) Immunofluorescence staining of paraffin-embedded CNVMs from a 78-year-old male patient with neovascular AMD. Sections were stained for αSMA (green), ZO-1 (red), type I collagen (type1 col; red), ROCK1 (green), and ROCK2 (green). Each row displays a pair of markers. From top to bottom: type1 col–ROCK1, type1 col–ROCK2, αSMA–ROCK1, αSMA–ROCK2, and αSMA–ZO-1. Within each row, the right panel shows the merged image. Increased αSMA and reduced ZO-1 expression were observed, along with strong type1 col expression. ROCK2-positive (green) cells co-localized with type1 col (red) in the merged images. Scale bars: 50 µm. (*B*) Higher-magnification merged images showing αSMA (green) with ROCK1 (green) or ROCK2 (green). Some αSMA-positive cells exhibited ROCK1/ROCK2 expression. Scale bars: 50 µm.

Supplementary Figure 2
